# Development of a predictive equation for resting energy expenditure in pediatric patients with oncological diagnosis

**DOI:** 10.3389/fnut.2025.1656975

**Published:** 2025-09-24

**Authors:** Alda Daniela García-Guzmán, Sandra Nayeli Becerra-Morales, Beatriz Adriana Pinzón-Navarro, Daffne Danae Baldwin-Monroy, Marta Zapata-Tarres, Liliana Velasco-Hidalgo, Azalia Avila-Nava, Rocío del Socorro Cárdenas-Cardos, Karla Maldonado-Silva, Martha Guevara-Cruz, Isabel Medina-Vera

**Affiliations:** ^1^Servicio de Oncología Médica, Instituto Nacional de Pediatría, Mexico City, Mexico; ^2^Tecnologico de Monterrey, Escuela de Medicina y Ciencias de la Salud, Mexico City, Mexico; ^3^Departamento de Metodología de la Investigación, Instituto Nacional de Pediatría, Mexico City, Mexico; ^4^Facultad de Medicina, Benemérita Universidad Autónoma de Puebla, Puebla, Mexico; ^5^Servicio de Gastroenterología y Nutrición Pediátrica, Instituto Nacional de Pediatría, Mexico City, Mexico; ^6^Unidad de Terapia Intensiva, Instituto Nacional de Pediatría, Mexico City, Mexico; ^7^Comisión Coordinadora de los Institutos Nacionales de Salud y Hospitales Federales de Referencia, Mexico City, Mexico; ^8^Hospital Regional de Alta Especialidad Península de Yucatán, Servicios de Salud del Instituto Mexicano del Seguro Social para el Bienestar (IMSS-Bienestar), Mérida, Yucatán, Mexico; ^9^Subdirección Hemato-Oncología, Instituto Nacional de Pediatría, Mexico City, Mexico; ^10^Departamento de Fisiología de la Nutrición, Instituto Nacional de Ciencias Médicas y Nutrición Salvador Zubirán, Mexico City, Mexico

**Keywords:** resting energy expenditure, cancer, pediatrics, equations, nutrition

## Abstract

**Background and aim:**

Pediatric cancer is a significant health concern, particularly in low- and middle-income countries with lower cure rates. The nutritional status of these patients is crucial because malnutrition, whether due to a deficiency or excess of energy, can negatively impact treatment response and long-term outcomes. Since resting energy expenditure (REE) is a key parameter for planning appropriate nutritional support, accurate assessment is essential. However, the most precise methods, such as indirect calorimetry (IC), are not always available, leading to predictive equations based on easily accessible variables. These equations may be inaccurate if they are not specifically designed for children with cancer. Therefore, this study presents an equation to estimate REE in pediatric patients with oncological diagnosis and to compare the accuracy of this equation with those of previous equations developed in different pediatric populations to assess its utility in a clinical population.

**Methodology:**

A cross-sectional study was conducted in pediatric patients aged 6 to <18 years with a recent oncological diagnosis. After diagnosis, anthropometric measurements were taken, nutritional status was assessed, body composition was determined using bioelectrical impedance, and REE was measured through IC.

**Results:**

A total of 226 pediatric participants were evaluated, of whom 203 were included in the final analysis. The majority had solid tumors (68.5%), followed by leukemia (20.2%) and brain tumors (11.3%). Significant differences in anthropometric and biochemical variables were observed among the different diagnoses, with patients with brain tumor having lower REE/kg of body weight. Two new REE prediction equations specific to this population were developed: the INP-simple model, which is based on basic clinical variables, and the INP-Morpho model, which includes body composition. Both new INP equations showed less bias in REE estimation (114.8, 95% CI: −408, 638) than traditional equations, including the Harris-Benedict (−133.6, 95% CI: −671.5, 404.2), FAO (−178.8, 95% CI: −683.9, 326.3), Schofield (−185.4, 95% CI: −697.6, 326.8), IOM (−201, 95% CI: −761.7, 359.7), Oxford (−110.6, 95% CI: −661.4, 440.1), Kaneko (−135.6, 95% CI: −652.5, 381.4) and Müller (−162.6, 95% CI: −715.1, 389.9) equations but not the Molnár equation (−82.3, 95% CI: −741.3, 576.7).

**Conclusion:**

Children with cancer often have energy expenditure levels that differ from the recommended values, increasing their risk of malnutrition or obesity. Predictive equations specifically developed for this population may offer improved accuracy for estimating REE in clinical settings, although external validation is still needed.

## Introduction

Pediatric cancer is among the leading causes of morbidity and mortality (1, 2); in high-income countries, more than 80% of affected children are cured, but in many low- and middle-income countries, the cure rate is only approximately 20% (3). However, with advances in treatments, survival rates have increased. The oncological process and associated therapies can have a significant effect on nutritional status (4), and this results in a critical challenge that could affect the response to treatment (5); since these are growing patients, it could affect long-term results (6) as well as early relapse in those with states of malnutrition (deficit or excess) (7), since a deteriorated nutritional status is associated with increased mobility and mortality, and accurate nutritional assessment followed by timely interventions could improve survival and clinical outcomes (8), significantly affecting quality of life (9, 10). Therefore, the role of nutrition (including nutritional assessment and interventions) during pediatric cancer treatment is extremely important and is reflected in patients’ clinical outcomes. Adequate nutrition, and thus a good nutritional status, reduces the total time needed to complete oncological treatment, decreases the need for antifungal therapy, and is associated with increased overall survival (11). A deteriorated nutritional status has frequently been observed at the time of diagnosis or during its subsequent management; malnutrition at the time of diagnosis has a high variability in prevalence, ranging from 7% in those with leukemia to 50% in those with neuroblastoma (12). Malnutrition is a critical challenge that can lead to poor treatment tolerance and poor prognosis; the causes are multifactorial but likely involve interactions between the iatrogenic consequences of treatment and complex interactions between energy and substrate metabolism; therefore, it is essential to address the nutritional status of pediatric patients with cancer as an integral part of their medical care, ensuring that they receive adequate nutritional support.

In pediatric patients with cancer, the interaction between systemic inflammation and metabolic alterations triggers cancer-related cachexia, which is characterized by a progressive loss of lean body mass with or without fat loss, resulting in an energy imbalance that compromises immune function and treatment response. The role of proinflammatory cytokines such as TNF-*α*, IL-1, and IL-6 in activating protein catabolism and mitochondrial dysfunction in muscle, processes that exacerbate physical and functional decline in these children (13), is highlighted in this work. The presence of cachexia in patients with an oncological diagnosis increases treatment-related toxicity and long-term morbidity and potentially affects mortality (14). The loss of muscle mass reduces the body’s capacity to metabolize drugs properly, while metabolic changes can alter drug clearance rates, making dose adjustment challenging (15). Moreover, immune dysfunction associated with cachexia impairs the patient’s ability to fight infections, increasing complications and prolonging hospital stays (16). These complications may contribute to treatment interruption and dose reduction, negatively influencing prognosis (17). Therefore, addressing malnutrition and cachexia early during the cancer treatment process is critical not only to improve nutritional status but also to enhance therapeutic outcomes and reduce adverse effects. Integrating nutritional support into the oncological care plan can improve quality of life and overall survival (18).

To receive adequate nutritional support, it is essential to evaluate the REE of patients to estimate their energy requirements in the nutritional context. Accurately determining REE in patients with cancer is crucial for nutritional planning, treatment optimization, and muscle mass preservation. Thus, REE is a key parameter in the nutritional care of patients with cancer since it directly influences their health, recovery, and quality of life (19). The most accurate method for measuring REE is indirect calorimetry (IC) (20); however, in many scenarios, this method is not available in the clinical context because it is costly (21) in the hospital and clinical context, and predictive equations (PEs) to estimate REE are frequently and quickly used because the information is obtained through easily accessible variables such as height, weight, age and sex, and fat-free mass (22). Many PEs have been developed over time. However, no specific REE prediction equation has been designed for the pediatric population with an oncological diagnosis (23).

The use of REE prediction equations may present biases due to individual variability, particularly when equations are used that were generated in a population different from the one to which they will be applied (14). Moreover, as a limitation, few equations have been validated explicitly in pediatric populations or in children with complex conditions such as cancer, which can lead to less precise results; having an equation designed for this population will help improve the accuracy of the estimation of energy expenditure and, therefore, the quality of nutritional management in pediatric patients with cancer. Therefore, the aims of the present study were to develop an equation to estimate REE in pediatric patients with oncological diagnosis, compare its accuracy with that of previous equations developed in different pediatric populations, and assess its utility in a clinical population.

## Materials and methods

### Study design

We performed a cross-sectional study of pediatric patients with recent (within 0 to 2 weeks) oncological diagnosis; participants were recruited between 2019 and 2024 in the Oncological Department of the Instituto Nacional de Pediatría, a third-level pediatric hospital in Mexico City, Mexico. Patients aged 6 to <18 years were included only if they were treatment-naïve, meaning that they had not yet started oncological treatment, and were excluded if they were taking medications known to affect metabolic function (insulin, corticosteroids or thyroid hormones) or antihistamines and herbal supplements, as well as if they had a diagnosis of hypothyroidism and hyperthyroidism. In addition, patients with severe cognitive or motor impairments that prevented completion of the required assessment, such as those with autism spectrum disorder or significant motor disabilities, were excluded.

After oncological diagnosis, anthropometric measurements were taken from the patients, and their nutritional status was evaluated. Body composition was also determined through electrical impedance and energy expenditure at rest with IC. Hand grip strength was evaluated with dynamometry, the level of physical activity was estimated, and the levels of routine serum biochemicals were taken from the clinical records.

### Clinical evaluations, anthropometric parameters, and nutritional status

All participants provided a medical history, in which the oncological diagnosis, date of diagnosis and clinical symptoms were documented. Participants were weighed on a calibrated digital scale (SECA 813; Seca GmbH&Co., Hamburg, Germany), and height was measured with an ultrasonic stadiometer (InLab S50; InBody Co., Seoul, Korea). Waist, hip, thigh, calf, wrist, and neck circumferences and mid-upper arm circumference (MUAC) were measured with a tape measure (SECA 201; Seca GmbH&Co., Hamburg, Germany). All measurements were taken with the patients standing up. Waist circumference was measured with the arms crossed in front of the chest; the measurement was taken between the lower edge of the 10th rib and the iliac crest. Hip circumference was measured by placing a tape measure at the largest protuberance of the buttock.

Thigh circumference was measured with the legs separated, and the tape was wrapped around the midpoint between the hip bone and the knee bone. Calf circumference was measured with the arms on the side of the body; the measurement was taken at the largest protuberance of the calf. Wrist circumference was measured with a tape measure without any pressure; the superior border of the tape was placed just distal to the prominence of the radial and ulnar bones. Neck circumference was measured with the upright and the head in the Frankfort horizontal plane; the tape was placed at the midpoint of the neck height. Finally, MUAC was measured with the arms on the side of the body; the tape was positioned halfway between the acromion and the radius.

Nutritional status was assessed using the BMI-for-age z score and height-for-age z score, according to the classification and values established by the WHO. AnthroPlus software (24) was used to obtain the BMI-for-age z score and height-for-age z score using data such as sex, date of birth, date of assessment, weight, and height. Reference points were classified according to the WHO, where < −3 SDs = severe malnutrition, −3 to −2 SDs = moderate malnutrition, ≥ − 2 to 1 SDs = standard, >1 to <2 SDs = overweight, and >2 SDs = obesity. The height-for-age indicator was assessed using the same data. The cutoff points were classified according to the WHO: 1.99 to −1.99 SDs = standard height, <−2 SDs = short, and >2 SDs = tall (25).

### Oncological diagnostic stratum

Oncological diagnoses were stratified into three groups: solid tumors (nasopharyngeal carcinoma, ganglioglioma, hepatoblastoma, lymphoma, rhabdomyosarcoma, synovial sarcoma, osteosarcoma, neuroblastoma, germ cell tumor, Ewing sarcoma, rhabdomyosarcoma, retinoblastoma, Wilms tumor, rhabdoid tumor, Langerhans cell histiocytosis, and hepatocarcinoma), leukemias (acute lymphoblastic leukemia and acute myeloid leukemia), and brain tumors (stem glioma, astrocytoma, ependymoma, and medulloblastoma).

### Measurement of resting energy expenditure

Resting energy expenditure by IC (REE-IC) was measured. We used CardioCoach VO2 max (Korr Medical Technologies Inc., Salt Lake City, Utah). The patients wore a face mask connected to the calorimeter, and a computer recorded variables such as VO2, FEO2, and FECO2. The patients were placed in a supine position for 10 min prior to the start of the test; after autocalibration with barometric pressure, temperature, and humidity, as well as during the respiration stabilization phase, the calorimeter analyzed the variables in a computer interphase every minute for 20 min (26, 27). Data from the software from patients with stable calorimetry analysis were defined as a respiratory coefficient between the physiological ranges [(QR) = 0.68–1.2] or having at least 1 period with less than 10% coefficient of variation (26).

### Body composition

Body composition was assessed by using a multifrequency bioimpedance device, employing bioelectrical impedance analysis (BIA) (InBody S10 R, InBody Co., Ltd., Seoul, Korea) with the standard technique; BIA’s internal equation was used. Measurements were performed with the patient in a supine position, with the arms separated from the trunk by ∼30° and the legs separated by ∼45°; there was no contact with the bed’s metal frame, and the room temperature was ambient. The patients had to lie in position for 5 min and were not allowed to eat or make any major physical effort in the preceding 8 h; they were also not allowed to drink in the preceding 3 h. Body weight and height were entered into the device. The area where the electrodes were to be placed was cleaned first with alcohol and then with electroconductive wet wipes of impedance equipment; the electrodes were placed on both the hands and the feet, according to the manufacturer’s instructions (InBody Co). The electrodes were kept in a sealed bag to protect against heat; the machine was calibrated before use with a known impedance circuit, per the manufacturer’s guidelines. The phase angle at 50 kHz was reported, and the following formula was used to determine it [Arc tangent (Xc/R)] Å ~ (180/5). The skeletal muscle mass index (SMI) was calculated by dividing skeletal muscle mass (kg) by the square of the height (m^2^).

### Handgrip strength

The posture for measuring the handgrip strength was standing, with legs straight and weight bearing, balanced on both feet, feet shoulder-width apart, shoulder adducted and neutrally rotated, elbow flexed to 90°, forearm in neutral position, wrist between 0° and 30° of dorsiflexion and between 0° and 15° of ulnar deviation; measurements were made with a Lafayette hydraulic hand dynamometer (Jamar Model J00105 Lafayette Instrument Company, United States) and were performed on the dominant hand in triplicate, and the average measurement was recorded (28).

### Estimation of physical activity

To determine the physical activity of the participants, 1 physical activity questionnaire was used; if the participant was under 14 years old, the PAQ-C was used, and for those over 14 years old, the PAQ-A was used (29). These questionnaires consisted of 9 and 10 items, each with a 5-point response scale ranging from low activity (score of 1) to high activity (score of 5). The level of physical activity was classified as low, moderate, or high based on the average scores obtained from the questionnaire (1–2.33: low, 2.34–3.66: moderate, and 3.67–5: high).

### REE predictive equations

The predictive equations evaluated in this study were selected because they are those used in the pediatric population: Food and Agriculture Organization/World Health Organization (FAO/WHO) (30), Schofield (31), Institute for Medicine of the National Academies and Food and Nutrition Board (IOM) (32), Oxford (33), Kaneko (34), and Müller (35). In addition, the new equations were compared with the Harris–Benedict equation (36), since it is the most common equation used, despite it being obtained from a population with normal body weight.

### Routine serum biochemistry

The most recent laboratory results of the following biochemical parameters were obtained from the medical records: albumin, creatinine, BUN (blood urea nitrogen), calculated urea, ALT (alanine aminotransferase), AST (aspartate aminotransferase), triglycerides, total cholesterol, HDL cholesterol (high-density lipoprotein), LDL (low-density lipoprotein) cholesterol, calcium, phosphorus, potassium and serum sodium, hemoglobin, leukocytes, hematocrit and mean corpuscular volume.

### Statistical analysis

Continuous variables are expressed as the means ± standard deviations or medians (25th–75th percentiles), and categorical variables are expressed as frequencies and percentages. The Kolmogorov–Smirnov test was used to assess the normality of the variables, and logarithmic transformation will be performed on those that do not have a normal distribution. To compare variables between oncological diagnoses, one-way ANOVA with *post hoc* Bonferroni’s multiple comparison test was used.

The correlation of each of the anthropometric variables, as well as age and sex, with the REE measured by IC was evaluated through Pearson or Spearman correlation depending on the distribution of the variables to analyze which of them had a greater correlation with the REE that could better predict the model. Subsequently, a stepwise regression analysis was carried out where we used those variables with an input significance less than 0.05 and an output probability of 0.10 as the criteria, where the REE measured by IC was taken as the dependent variable and age, sex, height, weight, fat-free mass and oncological diagnosis as independent variables, from which the best estimation model was obtained based on the R^2^ and the significance value.

The regression was then tested using the Intro method with the intention of identifying the variables with the greatest explanatory power and statistical significance, and a multiple linear regression was carried out considering the biological relationship of the independent variables introduced into the model. To validate the multiple linear regression model, several statistical assumptions were evaluated. Homoscedasticity was assessed using plots of residuals versus fitted values. Multicollinearity was checked by calculating the VIF for each independent variable. Independence of errors was evaluated using the Durbin–Watson statistic, which indicated no significant autocorrelation. These analyses confirmed the validity of the model.

Categorical variables were coded as follows: sex was treated as a binary variable (0 = girls. 1 = boys), although for clarity, separate equations were presented for each sex. Oncological diagnosis was included, and although total REE did not significantly differ across oncological diagnoses, relevant differences emerged when REE was adjusted by body weight (REE/body weight). In this analysis, patients with solid tumors and leukemia presented similar REE/body weight values, whereas those diagnosed with brain tumors presented significantly different values. Based on these findings, oncological diagnosis was included as an independent variable in the statistical model. From a statistical standpoint, the inclusion of this variable improved the model’s explanatory capacity, as evidenced by an increase in the adjusted R^2^ value. Clinically, the observed metabolic differences across tumor types further justified the incorporation of oncological diagnosis in the predictive equations for REE. This variable was included using dummy coding with leukemia as the reference category (OD = 0), solid tumors coded as OD = 1, and brain tumors coded as OD = 2. The numeric values for the OD in the final equations represent the regression coefficients associated with each category. Bland–Altman’s method was used to evaluate the agreement between the REE-IC and the REE-PE estimated by the new equation by plotting the distribution of the differences between the REE-PE and REE-IC (mean bias and 95% CI) against their respective average values. The same procedure was performed with the other PEs from the literature. To evaluate the accuracy of the new predictive equations and the previously reported REE equations in oncology patients, the mean absolute error (MAE) was used as the primary performance metric.

Patients with missing data were excluded from the analysis, and no data imputation was performed for the variables. All *p* values were two-tailed, and we considered *p* < 0.05 to indicate statistical significance. All the statistical analyses were performed using SPSS (version 25; SPSS, Inc., Chicago, IL) and GraphPad Prism (version 9.0; GraphPad Software, Inc., San Diego, CA) software. The sample size was calculated with a coefficient correlation equation (37) to obtain an optimal number of subjects to develop a new model for predicting REE. Ten variables were considered for the sample size assessment. The size of the sample required to develop the equation was 200 subjects. The relationships between REE and the ten variables were assessed using Pearson’s correlation coefficients.

## Results

**Figure 1:**
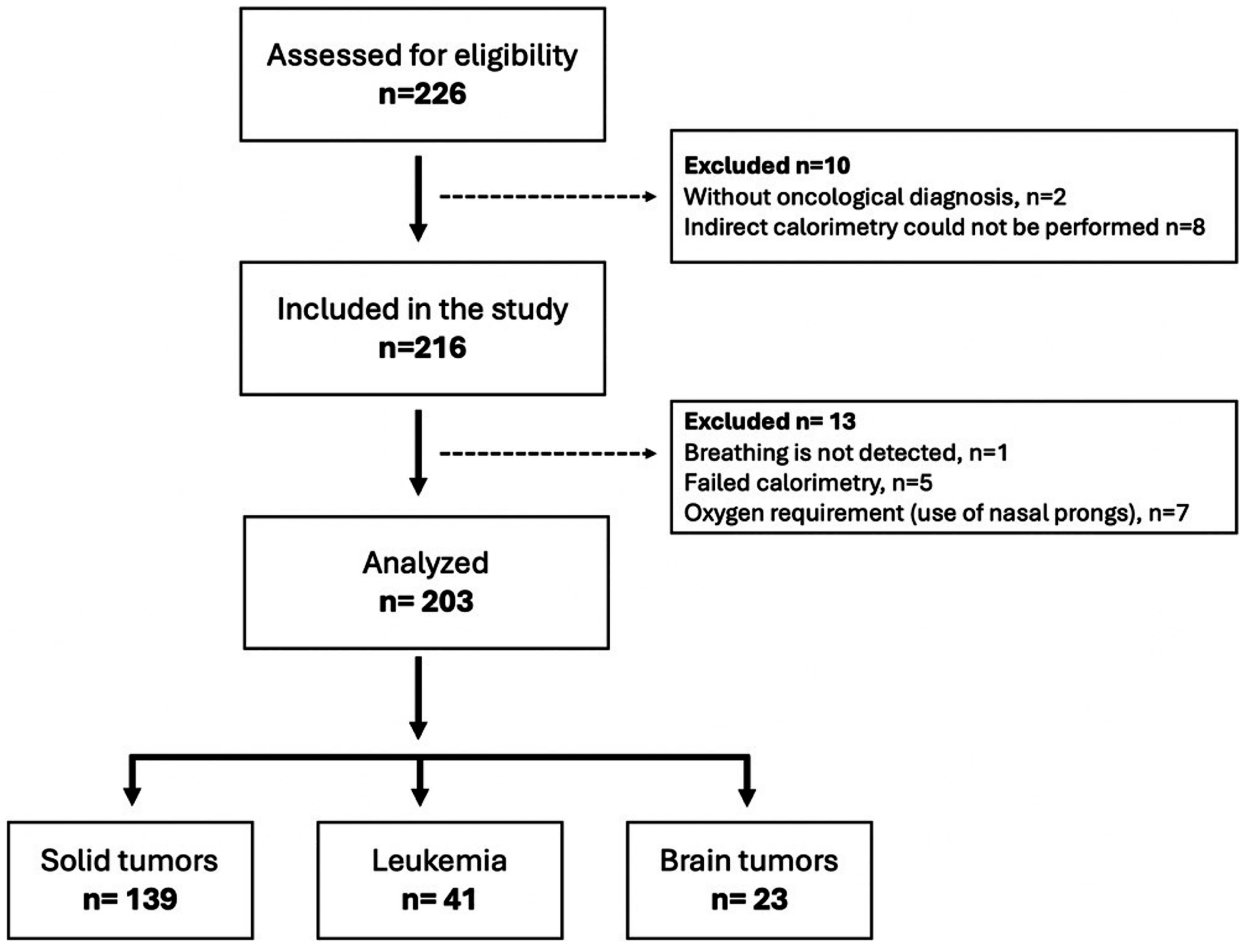
Flow chart of the study participants.

A total of 226 participants were evaluated, of whom 2 were excluded because they did not have an oncological diagnosis at the time of confirmation. Among those who met the inclusion criteria, 8 could not undergo IC assessment because the mask was too big for their face or because they moved too much to perform the test, and 7 could not wear the mask because they had an oxygen requirement and used nasal cannulas. Of the 209 who did undergo the IC assessment, the data from one were not used because breathing could not be detected during the test, and the data from 5 a variation in the O_2_ volume > 10% were not stable. Thus, the data from 203 participants were analyzed, of whom 68.5% had a diagnosis of a solid tumor, 20.2% of leukemia and 11.3% of a brain tumor, as shown in Figure 1.

### Baseline characteristics of the participants

The anthropometric variables, body composition characteristics and REE of the participants over all oncological diagnosis strata are shown in Table 1, and we observed that 57.6% of the population were men, with an average age of 12.2 ± 3 years. Analysis of the diagnostic strata revealed that the variables of waist circumference and body fat percentage significantly differed among children with a diagnosis of a brain tumor. Although the estimated visceral fat area was not significantly different, the average was significantly different (74.6 ± 50.7 cm^2^) from the other two oncological strata analyzed (solid tumor: 56.3 ± 41.8 cm^2^; leukemia: 51.3 ± 37.6 cm^2^). All participants had an average fat-free mass of 33.8 ± 11 kg and a body cell mass of 21.9 ± 7.4 kg. The average phase angle was 5.2 ± 2.2°, and although no statistically significant differences were observed, the cerebral tumor stratum had an average of 4.9 ± 1.2°, the solid tumor stratum had an average of 5.1 ± 1.2°, and the leukemia stratum had an average of 5.7 ± 4.3°.

**Table 1:** Characteristics of all the subjects and comparison between diagnosis oncological strata.

Variables	All *n* = 203	Solid tumor *n* = 139	Leukemias *n* = 41	Brain tumor *n* = 23	*p* value
Age (years)	12.2 ± 3	12.4 ± 2.9	11.3 ± 3.3	13 ± 2.8	0.06
Sex boys/girls (%)	57.6/42.4	54.7/45.3	58.5/41.5	73.9/26.1	0.222
Body weight (kg)	45.7 ± 16.6	45.5 ± 16.3	43.8 ± 17.3	50.4 ± 17.3	0.297
Height (cm)	147.6 ± 23.6	147 ± 26.1	147.5 ± 18.5	151 ± 14.9	0.649
BMI-age (z score)	0.64 ± 1.5	−0.071 ± 1.6	0.234 ± 1.2	0.561 ± 1.3	0.149
Height-for-age (z score)	−0.404 ± 1.04	−0.471 ± 1.08^a^	−0.044 ± 0.87^b^	−0.644 ± 0.975^a^	0.035
Waist circumference (cm)	71.8 ± 12.5	70.8 ± 11.92^b^	71.5 ± 12.79^b^	78.16 ± 14.7^a^	0.039
Hip circumference (cm)	78.5 ± 13.5	78.3 ± 13.40	77. 4 ± 14.3	82.0 ± 13.5	0.431
Neck circumference (cm)	31.9 ± 4.3	31.9 ± 4.0	30.6 ± 4.8^b^	33.8 ± 4.2^a^	0.015
Thigh circumference (cm)	40.0 ± 8.4	40.4 ± 8.2	38.0 ± 9.2	41.3 ± 7.9	0.197
Calf circumference (cm)	28.8 ± 5.7	28.8 ± 5.5	28.9 ± 6.4	29.1 ± 5.6	0.974
MUAC (cm)	22.6 ± 4.5	22.6 ± 4.5	21.6 ± 4.5	24.2 ± 4.4	0.088
Body composition					
Fat mass (%)	23.4 ± 11	23.3 ± 11^b^	20.9 ± 10^b^	29.2 ± 9^a^	0.020
Fat-free mass (kg)	33.8 ± 11	34 ± 11	32.8 ± 11	34.1 ± 11	0.819
Intracellular water (L)	15.3 ± 5.1	15.4 ± 5.2	14.8 ± 5.1	15.3 ± 5	0.806
Extracellular water (L)	9.4 ± 3.1	9.4 ± 3.1	9.1 ± 3.2	9.6 ± 3.1	0.827
Total body water (L)	24.7 ± 8.2	24.8 ± 8.2	23.9 ± 8.2	24.9 ± 8.0	0.822
Proteins (kg)	6.61 ± 2.2	6.68 ± 2.2	6.42 ± 2.1	6.62 ± 2.1	0.812
Minerals (kg)	2.48 ± 0.7	2.49 ± 0.7	2.42 ± 0.7	2.58 ± 0.8	0.710
Skeletal muscle mass (kg)	17.9 ± 6.7	18.1 ± 6.8	17.3 ± 6.6	17.9 ± 6.6	0.811
Body cell mass (kg)	21.9 ± 7.4	22.1 ± 7.5	21.2 ± 7.3	21.9 ± 7.2	0.807
Visceral fat area (cm^2^)	57.2 ± 42.3	56.3 ± 41.8	51.3 ± 37.6	74.6 ± 50.7	0.109
Total phase angle (°)	5.2 ± 2.2	5.1 ± 1.2	5.7 ± 4.3	4.9 ± 1.2	0.275
Indirect calorimetry					
REE (kcal/day)	1,180 ± 319	1,198 ± 316	1,179 ± 354	1,067 ± 246	0.214
REE/body weight (kcal/kg/day)	28.4 ± 9.3	28.7 ± 9.1^a^	29.8 ± 10^a^	23.4 ± 7.1^b^	0.026
REE/FFM (kcal/kg/day)	37.2 ± 10.8	37.6 ± 10.8	38.5 ± 11.4	32.4 ± 8	0.111
VO_2_ consumption (ml/min)	171 ± 47.6	129.1 ± 36.3	174.1 ± 54.7	151 ± 37.3	0.172
VCO_2_ consumption (ml/min)	128.1 ± 39.6	129.1 ± 36.3	133.8 ± 52.4	109. 9 ± 26.7	0.112
RQ	0.74 ± 0.10	0.74 ± 0.09	0.73 ± 0.13	0.72 ± 0.12	0.829
Physical activity					
Physical activity (points)	1 (1–1.45)	1 (1–1.44)	1 (1–1.57)	1 (1–1.59)	0.899
Physical activity level, *n* (%)					0.851
Mild	184 (90.6)	127 (91.2)	36 (87.8)	21 (91.3)	
Moderate	17 (8.4)	10 (7.2)	5 (12.2)	2 (8.7)	
High	2 (1)	2 (1.6)	0 (0)	0 (0)	

BMI-age: body mass index-for-age; MUAC: Mid-Upper Arm Circumference; REE: Resting Energy Expenditure; REE/FFM: Resting Energy Expenditure per Fat-Free Mass; VO_2_: Oxygen Consumption, VCO_2_: Carbon Dioxide Production; RQ: Respiratory Quotient.

With respect to the level of physical activity performed, no statistically significant differences were found between diagnostic strata. However, 90.6% of all participants reported mild physical activity, and only 1% (*n* = 2) reported a high level of physical activity. As indicated by the profile of biochemical variables, the leukemia diagnosis stratum presented a higher level of BUN than solid tumors and brain tumors did (*p* = 0.001). With respect to triglycerides, the solid tumor stratum presented a lower level than the leukemia and brain tumor strata did (*p* = 0.038); interestingly, the leukemia stratum presented lower hemoglobin (*p* = 0.001) and hematocrit (*p* = 0.001) concentrations, and the other biochemical variables are shown in Supplementary Table 1. When the biochemical variables were correlated with REE, no significant associations were found for most of them, with the exception of creatinine, whose correlation was r = 0.370 (*p* = 0.001); urea, r = 0.215 (*p* = 0.011); and leukocytes, r = 0.271 (*p* = 0.001).

For the variables of REE, no statistically significant differences were observed in total REE between diagnostic strata. However, when the REE/kg of body weight was analyzed, the REE/kg of body weight (23.4 ± 7.1 kcal/kg/day) was lower in the brain tumors than in the solid tumor stratum (28.7 ± 9.1 kcal/kg/day) and leukemia (29.8 ± 10 kcal/kg/day) (*p* = 0.026). The correlations between all the anthropometric and body composition variables and REE are shown in Table 2. Among the anthropometric variables, body weight was the most strongly correlated (r = 0.586, r^2^ = 0.343; *p* < 0.001). Among the body composition variables, fat-free mass was most strongly correlated (r = 0.577; r^2^ = 0.332; *p* < 0.001), and among the clinical variables, age was greatest (r = 0.437; r^2^ = 0.190; *p* < 0.001).

**Table 2:** Correlation coefficients for REE measured by IC and anthropometric and body composition variables.

Variables	R	R^2^	*p* value
Anthropometric variable			
Body weight (kg)	0.586	0.343	<0.001
Height (cm)	0.154	0.023	0.030
BMI-age (z score)	0.303	0.092	0.001
Height-for-age (z score)	0.089	0.007	0.212
Waist circumference (cm)	0.499	0.294	<0.001
Hip circumference (cm)	0.488	0.238	<0.001
Neck circumference (cm)	0.514	0.264	<0.001
Thigh circumference (cm)	0.486	0.236	<0.001
Leg circumference (cm)	0.562	0.315	<0.001
MUAC (cm)	0.497	0.247	<0.001
Clinical variables			
Age (years)	0.437	0.190	<0.001
Sex	−0.198	0.4	0.005
Body composition variables			
Fat mass (%)	0.388	0.150	<0.001
Fat-free mass (kg)	0.577	0.332	<0.001
Intracellular water (L)	0.571	0.326	<0.001
Extracellular water (L)	0.570	0.324	<0.001
Total body water (L)	0.576	0.331	<0.001
Proteins (kg)	0.570	0.324	<0.001
Minerals (kg)	0.569	0.323	<0.001
Skeletal muscle mass (kg)	0.571	0.326	<0.001
Body cell mass (kg)	0.571	0.326	<0.001
Visceral fat area (cm^2^)	0.299	0.089	<0.001
Total phase angle (°)	0.109	0.011	0.139

BMI-age: body mass index-for-age; MUAC: Mid-Upper Arm Circumference.

### Equation development for pediatric patients with an oncological diagnosis

Two REE prediction equations were developed for pediatric patients with an oncological diagnosis. The first was easily obtainable variables for the clinical context, and we call it the INP-simple model, which includes weight in kilograms, age in years, height in centimeters, sex, and oncological diagnosis. The other model was developed with a body composition variable, and we called it the INP-Morpho model, which was developed with weight in kilograms, age in years, free fat mass in kilograms, height in centimeters, sex and oncological diagnosis; the new equations are presented in Table 3.

**Table 3:** Resting energy expenditure prediction equation developed for Mexican pediatric with oncological diagnosis.

Simple REE equation (INP-simple model)		Morphofunctional REE equation (INP-Morpho model)	
Girls: REE = (11*W) + (22*A) – (3*H) + (OD) + 640	R = 0.660	Girls: REE = (9*W) + (17*A) + (4*FFM) – (3*H) + (OD) + 650	R = 0.664
	R^2^ = 0.435		R^2^ = 0.441
Boys: REE = (11*W) + (22*A) – (3*H) + (OD) + 520	*p* = 0.0001	Boys: REE = (9*W) + (17*A) + (4*FFM) – (3*H) + (OD) + 540	p = 0.0001
W: weight (kg); A: age (y); H: height (cm) Substitute the value of Oncological diagnosis (OD) in the equation according to the patient’s diagnosis: *Brain tumor + 95* *Solid tumor + 195* *Leukemia + 219*		W: weight (kg); A: age (y); FFM: fat free mass; H: height (cm) Substitute the value of Oncological diagnosis (OD) in the equation according to the patient’s diagnosis: *Brain tumor + 100* *Solid tumor + 201* *Leukemia + 225*	

*Sex was treated as a binary variable in both equations. Since the equations are presented separately for girls and boys, the sex coefficient has been absorbed into the intercept term.

### Equation validation in pediatric patients with oncological diagnosis

**Figure 2:**
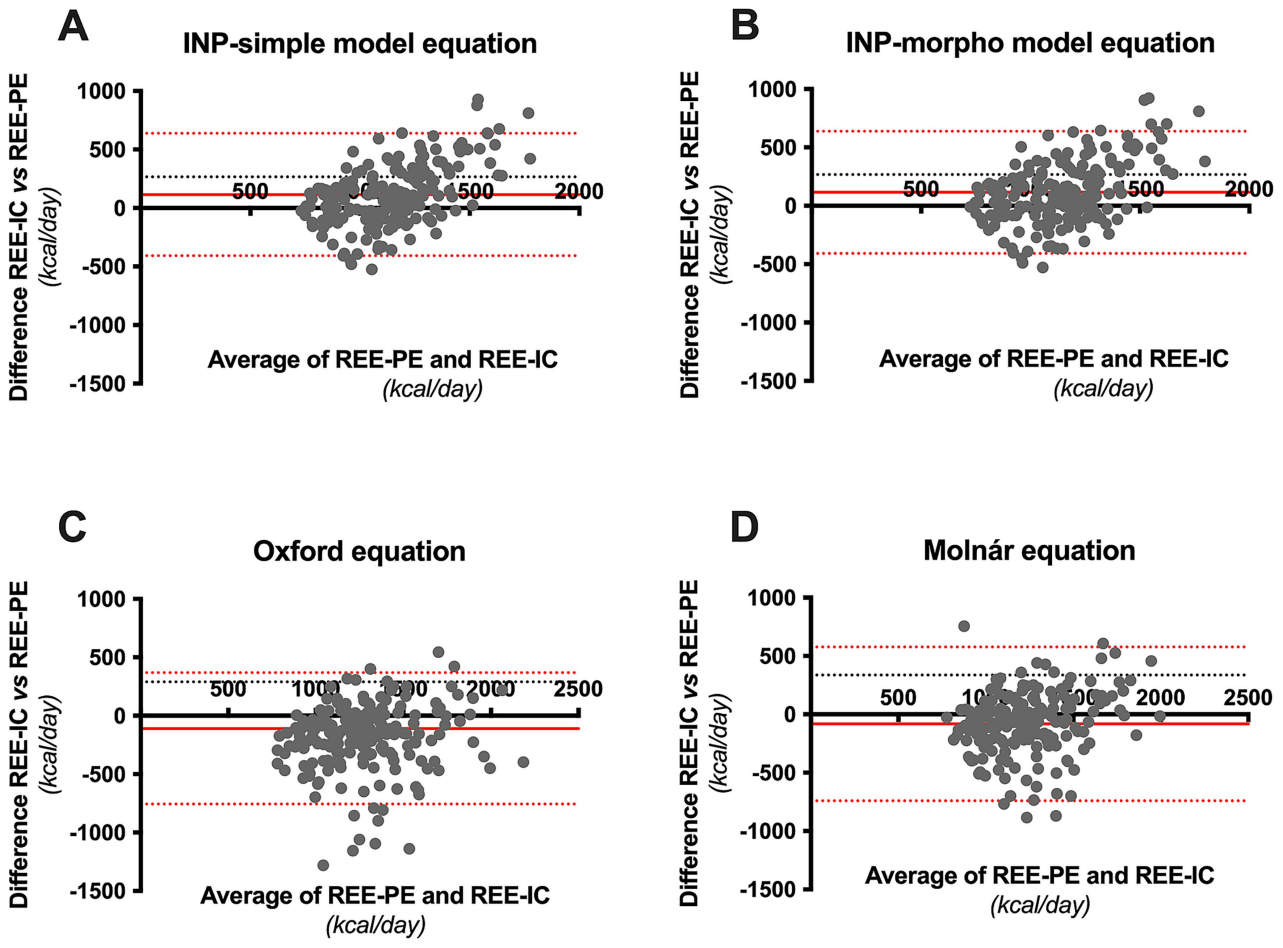
Bland–Altman plots displaying the agreement and difference between the resting energy expenditure by indirect calorimetry (REE-IC) and the resting energy expenditure predicted (REE-PE) by the **(A)** simple model, **(B)** morpho model, **(C)** Oxford equation and **(D)** Molnár equation. The solid red line represents the mean bias of the prediction equation, the red dotted lines represent the limits of agreement (± 95% CI), and the black dotted lines represent the ±10% accuracy limits.

The average REE measurement by IC was 1,200 ± 306 kcal/day; however, compared with the REE prediction equations, a lower bias was observed with the new equations designed, and with the Molnár equation, the equation that represented a greater bias for this population was that of IOM and Schofield (Figure 2). In the validation of the predictive equations for REE, the equations with the lowest MAEs were the Morpho model (161 kcal), the simple model (168.5 kcal), and the Molnár equation (166.8 kcal); these equations showed, on average, smaller absolute deviations than the REE measured by IC. In contrast, classical equations such as Schofield (244.5 kcal), Müller (228.9 kcal), and IOM (200.2 kcal) presented the highest MAEs (Table 4).

**Table 4:** Equations validation in pediatric with oncological diagnosis.

Predictive equations	REE mean kcal	Bias (CI 95%) kcal/day	Mean absolute error kcal
Indirect calorimetry	1,200 ± 306		
Simple model	1,086 ± 196	114.8 (−408, 638)	168.5 (82.2–345.7)
Morpho model	1,088 ± 191	114.8 (−408, 638)	161 (84.5–340)
Harris-Benedict	1,334 ± 269	−133.6 (−671.5, 404.2)	196 (85.3–332.1)
FAO	1,379 ± 275	−178.8 (−683.9, 326.3)	209.6 (97.5–359.5)
Schofield	1,385 ± 287	−185.4 (−697.6, 326.8)	244.5 (103.7–413.9)
IOM	1,400 ± 316	−201 (−761.7, 359.7)	200.2 (106.5–383.7)
Oxford	1,311 ± 324	−110.6 (−661.4, 440.1)	194 (93.3–347.3)
Kaneko	1,336 ± 243	−135.6 (−652.5, 381.4)	183.7 (97.5–337.6)
Molnár	1,281 ± 303	−82.3 (−741.3, 576.7)	166.8 (68.1–298.3)
Müller	1,364 ± 248	−162.6 (−715.1, 389.9)	228.9 (111.3–368.2)

Bland–Altman’s method was used to evaluate the agreement between the resting energy expenditure measured by indirect calorimetry (REE-IC) and the predicted resting energy expenditure (REE-PE) estimated by all the equations by plotting the distribution of the differences between REE-PE and REE-IC (mean bias).

## Discussion

Accurate estimation of energy requirements in children with cancer is important for nutritional management. When this measurement is not feasible or IC is not available, prediction equations become valuable tools for estimating REE. In our study, two equations were designed to estimate REE in children with cancer. The first, called the INP-simple model, uses easily obtainable variables such as weight, age, height, sex, and type of cancer diagnosis. The second, called the INP-morpho model, can be applied if body composition analysis equipment is available and includes fat-free mass and the variables in the simple model. These are the first equations created with accessible data to estimate energy requirements in children with cancer and are especially useful in places where IC is unavailable. The equation explains between 43 and 44% of the variability in the REE, meaning that nearly half of the variation in the data can be accounted for; this indicates a moderate level of precision; therefore, the model has an acceptable predictive capacity. However, this finding also suggests that there may be other important factors not included in the equation or a high degree of random variability.

This study also compared the accuracy of different equations and revealed that the designed equations and the Oxford equation showed the least bias. The Oxford equation, created in a pediatric population in England, also uses body weight, age, and sex as its main variables. On the other hand, predictive equations are easy to use, but some equations have limitations. They do not consider important factors such as body composition or the impact of energy on chronic disease (38). Similarly, several studies have shown that prediction equations are unreliable for assessing nutritional needs in children with chronic diseases (39, 40). These studies indicate that although predictive equations are the most economical and rapid method for estimating REE, they lack the reliability necessary to measure REE, which calls into question their usefulness in the clinical setting. Although CI is the ideal method, fewer than 10% of centers that treat children with cancer have this equipment. Therefore, understanding the accuracy of prediction equations in these patients is important since an incorrect estimate of energy requirements and inaccurate energy supply negatively affect growth and development and exacerbate other known negative outcomes associated with malnutrition (41, 42). It is therefore important that this study demonstrated the accuracy of certain previously designed equations and the design of new equations in children with cancer. Similarly, in another study, Kellerman and colleagues determined the impact of chemotherapy exposure on REE in children with a recent cancer diagnosis during the first 6 months of intensive chemotherapy and whether predictive equations allowed for the estimation of the accuracy of these requirements at the time of diagnosis as a basis for nutritional interventions. Their findings illustrate the inability of commonly used predictive equations to calculate REE at the time of childhood cancer diagnosis. While a general overestimation, significant bias, and moderate to low agreement were observed for all three equations, the WHO and Schofield (weight, height) equations were more reliable for resting energy estimates than the RDA equation was (43). Our study agrees with the overestimation of the Schofield and FAO equation.

In the population studied, a greater percentage of fat mass was observed in patients with brain tumors, as well as a lower REE/body weight ratio, than in children with leukemia or solid tumors. The difference is between 5 kcal/kg/day. This could be due to hypothalamic involvement. In a retrospective cohort of children with brain tumors at risk for hypothalamic dysfunction, approximately 67% had a measured REE less than 90% of their predicted REE, which was linked to the severity of hypothalamic damage, indicating a reduction in resting energy expenditure (44). In contrast, patients with brain tumors had higher body weights. This finding is documented, with 13 to 40% of these patients being overweight or obese, mainly due to hormonal alterations and hypothalamic damage, rather than the tumor directly causing obesity (45, 46). In these patients, adipose tissue may exhibit reduced thermogenic activity, which facilitates fat accumulation (47).

However, it is also important to consider the type of tumor referred to in the equation we designed since it has previously been demonstrated that regardless of tumor size, small tumors experience high rates of glycolysis and lactate production, independent of their oxygen supply (48), and excess lactate is converted back into glucose in the liver (cyclodeoxyribose), which leads to a net consumption of adenosine triphosphate (49, 50). This increase in glucose turnover may contribute significantly to high REE and muscle catabolism in patients with cancer (51, 52). In terms of the variables most closely related to REE in our study, the results were like those reported in previous research conducted on adults. In this study, 714 patients with cancer and 642 healthy individuals were evaluated, and REE and body composition were analyzed to determine their relationships with energy expenditure. The results revealed that patients with cancer had an elevated REE (47%). Similarly, the type of cancer, pathological stage, and duration of the disease influenced the REE. In contrast, fat mass, fat-free mass, and body cell mass decrease in patients with cancer, which may be related to elevated REE (53).

In our equation, we also found that age has a significant influence on REE. Age is a significant predictor of REE in patients with cancer, and advanced age is generally associated with lower REE when adjusted for body composition. However, most of the available data focus on adults, and direct pediatric data are limited. In adult patients with cancer, age, fat-free mass (FFM), and inflammation (measured by C-reactive protein, CRP) together explain much of the variability in REE, suggesting that similar factors may influence pediatric patients as they age (54, 55). Another important variable in the design of the equations that influenced the REE was sex. Sex differences in energy expenditure are evident in children, as compared with girls, boys tend to have higher resting and total energy expenditure, mainly due to higher activity levels and intrinsic factors beyond body composition. Fat-free mass is the main primary determinant of energy expenditure, but sex remains an independent predictive factor. However, when body composition is very similar, these differences may diminish. Understanding these patterns is important for tailoring nutritional and physical activity recommendations to children (56–60).

Studies have shown that patients with cancer are malnourished or overfed, according to the use of predictive equations, compared with the use of IC (61, 62); this is important in pediatric patients with cancer, as both underweight and overweight can negatively affect their clinical outcome and exacerbate the late effects of treatment. Malnutrition can lead to impaired growth and development, increased infection rates, increased use of resources, poor therapeutic response, and long hospital stays. In addition, children and adolescents who are malnourished during their illness are at greater risk of morbidity and mortality. It is therefore essential to identify pediatric patients at risk of developing malnutrition and to ensure the reliability of the equations used to determine their REE.

Additionally, overeating can predispose patients to developing hyperglycemia and liver dysfunction, which can cause fluid overload. In addition, children with certain types of cancer are at greater risk of becoming overweight or obese during treatment due to the therapies used (19). Overeating can exacerbate weight gain, increasing the likelihood of developing obesity-related complications that persist during survival (63). A study by Zhang et al. evaluated the REE in childhood cancer survivors and reported that it was almost 500 kcal/day lower than the estimated energy requirements. These findings suggest that obesity in this patient population could be related to a reduction in total energy expenditure (64). Therefore, it is essential to know the REE of a child with cancer to promote recovery and healing and to prevent or slow the progression of malnutrition (65).

We use the KORR Ree Vue calorimeter, which, we emphasize, has been previously evaluated in studies of overweight and obese adolescents and has been shown to be a reliable and accurate assessment tool compared to traditional IC, unlike other portable indirect calorimeters (66). Adjusting energy intake in patients with cancer at the beginning of treatment is essential, as adequate caloric intake improves nutritional status, helps maintain body weight, and is associated with better clinical outcomes and lower mortality (67, 68). However, predictive equations for REE are also necessary during treatment, since energy requirements can vary significantly throughout the course of the disease and its management. Changes in metabolism, body composition, and the effects of chemotherapy or radiotherapy can alter actual energy expenditure both during and after treatment (69).

The heterogeneity of different oncological diagnoses and medical treatments could have a significant impact on REE. Solid tumors may induce inflammatory responses that differ from those observed in leukemias, potentially altering REE in distinct ways. Additionally, compared with other agents, certain chemotherapeutic agents have more pronounced catabolic effects and can modify basal metabolism. Although patients receiving corticosteroids were excluded from the present study to avoid their confounding effects on metabolism and REE, this methodological decision also limits the applicability of the findings to real-world clinical settings, where corticosteroids are commonly used, particularly in leukemia treatment. Therefore, caution should be exercised when these results are generalized to patients undergoing active treatment. In this context, clinical and therapeutic differences could contribute to the variability in REE and should be considered in future studies. A longitudinal design would be particularly relevant for evaluating the evolution of REE throughout treatment and recovery, as well as the inclusion of larger and more homogeneous samples in terms of tumor type and treatment protocol. Such an approach would allow for a better understanding of the determinants of REE and, in turn, help optimize nutritional support with the aim of improving clinical outcomes in pediatric oncology. In parallel, the analysis of biochemical variables in this study revealed that most were not significantly correlated with REE, suggesting that other physiological or pathological factors may influence energy metabolism. However, moderate positive correlations were observed with creatinine (r = 0.370, *p* = 0.001), calculated urea (r = 0.215, *p* = 0.011), and the leukocyte count (r = 0.271, *p* = 0.0001). The association with creatinine may reflect a relationship between REE and muscle mass or renal function, while elevated urea levels could indicate increased protein catabolism. The correlation with leukocytes supports the hypothesis that inflammatory processes may increase REE in these patients. Nevertheless, these associations, while statistically significant, are of modest magnitude and should be interpreted with caution. Further research is needed to clarify the mechanisms linking clinical biomarkers with the metabolic alterations observed in this population.

Among the limitations of the study are the imbalance in the diagnostic stratum groups, where more patients were diagnosed with solid tumors, followed by leukemia and brain tumors, in addition to the diversity of diagnoses within the proposed strata. Additionally, tumor staging was not included in the analysis due to lack of consistent data across participants. This variable may influence energy metabolism and should be considered in future studies. Another limitation is that the data obtained in this study belong to a single group of patients from one hospital. Although it is a significant sample size, the fact that these data are from a single center may affect the generalizability of the results. However, this is the first study to design an equation for children with cancer. Another limitation is that a broader range of variables, such as the type and severity of the disease, were not included. Another limitation is that the equation overestimates the REE by an average of 115 kcal compared with the measured value; this reflects a positive bias, indicating a systematic rather than a random error. While this level of bias is generally considered acceptable, it could theoretically lead to a weight gain of approximately 5 kg over the course of a year (70). Nonetheless, it is important to note that this equation demonstrates a smaller bias than the other equations evaluated. One important limitation of our study is the exclusion of patients who were unable to complete the assessments due to severe cognitive or motor impairments, including those with autism spectrum disorder or significant motor disabilities. While necessary to ensure the integrity of the data collected, this exclusion may introduce selection bias and limit the generalizability of our findings to the broader pediatric oncology population. Future studies should consider alternative assessment strategies to include children with such conditions to ensure more inclusive and representative data.

A strength of this study is that body composition was included in the design of the equation, given that lean body mass is directly related to energy requirements. Another strength is that newly diagnosed patients who had not started treatment were included. Therefore, it would be worthwhile to explore further how variables influence the measurement of REE in different contexts. Moreover, the evidence of our results should be reinforced with a larger sample size and validation of the equations, and changes across different phases of treatment, follow-up, and disease severity should be determined.

## Conclusion

Children with cancer tend to have energy expenditures that are lower or higher than the recommended level depending on the oncological diagnosis, which increases their risk of obesity or malnutrition. The use of predictive equations tailored to this population is important for accurately estimating REE in clinical settings. This study supports the use of equations specifically developed for children with cancer, as they appear more appropriate than standard equations. However, it is crucial to note that these new equations should be applied only in children diagnosed with cancer, considering those who have not yet started oncological treatment or other medications such as corticosteroids. Additionally, the type of cancer should be considered, as it significantly influences energy expenditure. Further studies are still needed to refine these predictive models and to identify specific markers that explain variations in REE across different cancer types. Standard predictive equations may not accurately estimate individual energy needs, highlighting the importance of personalized nutritional assessment and continuous research in this area.

## Data Availability

The raw data supporting the conclusions of this article will be made available by the authors, without undue reservation.
