# Supplementary material for: Development of a predictive equation for resting energy expenditure in pediatric patients with oncological diagnosis
Source: Front Nutr. 2025 Sep 24;12:1656975. doi: 10.3389/fnut.2025.1656975 (PMC12504883; doi:10.3389/fnut.2025.1656975)
Supplement: Supplementary file 1 [file Table_1.docx]

***SUPPLEMENTARY TABLES***

| **Supplementary table 1.** Biochemical variables | | | | | |
| --- | --- | --- | --- | --- | --- |
|  | **All**  **n= 203** | **Solid tumor**  **n=139** | **Leukemias**  **n=41** | **Brain tumor**  **n=23** | **p value** |
| Albumin, *g/dL* | 4.06 ± 0.6 | 4.1 ± 0.6 | 3.9 ± 0.5 | 3.90 ± 0.7 | 0.434 |
| Creatinine, m*g/dL* | 0.53 ± 0.2 | 0.54 ± 0.1 | 0.5 ± 0.1 | 0.55 ± 0.17 | 0.746 |
| BUN, m*g/dL* | 12.7 ± 5.5 | 11.9 ± 4.6^b^ | 17.3 ± 7.7^a^ | 11.9 ± 4.9^b^ | 0.001 |
| Calculated urea, *mg/dL* | 27.1 ± 11.3 | 25.7 ± 9.8 ^b^ | 34.7 ± 14.8^a^ | 24.8 ± 10.7 ^b^ | 0.002 |
| ALT, *UI/L* | 18.0 (11, 36.5) | 17.5 (11, 34.5) | 18.0 (11.0-70.0) | 22.5 (11.7, 34.7) | 0.819 |
| AST, *UI/L* | 22.0 ( 17, 28.7) | 22.0 (17, 30) | 22.0 (16.5,51.5) | 20.0 (14.7, 26.0) | 0.453 |
| Triglycerides, *mg/dL* | 131 ± 76.6 | 114 ± 47.5^b^ | 159 ± 95^a^ | 147 ± 45.5^a^ | 0.038 |
| TC, *mg/dL* | 146.0 ± 39.9 | 143.9 ± 38.7 | 140.4 ± 43.8 | 164.7 ± 40.8 | 0.314 |
| HDL-C, *mg/dL* | 38.4 (29.4, 49.5) | 41.0 (31.5, 50.4) | 29.6 (18.9, 52.4) | 40.9 (36.1, 42.3) | 0.306 |
| LDL-C, *mg/dL* | 81.4 ± 31.9 | 80.3 ± 31.5 | 70.2 ± 35.0 | 97 ± 19 | 0.209 |
| Calcium, *mg/dL* | 9.0 ± 1.1 | 9.1 ± 1.1 | 8.7 ± 1.0 | 9.0 ± 1.5 | 0.235 |
| Phosphorus, *mg/dL* | 4.5 ± 1.8 | 4.6 ± 2.1 | 4.3 ± 1.1 | 4.3 ± 0.89 | 0.624 |
| Potassium, *mmol/L* | 4.0 ± 0.40 | 4.1 ± 0.40 | 3.9 ± 0.47 | 3.9 ± 0.26 | 0.068 |
| Sodium, *mmol/L* | 137.4 ± 2.7 | 137.3 ± 2.5 | 137.1 ± 3.2 | 138.2 ± 3.2 | 0.324 |
| Hemoglobin, *mmol/L* | 12.0 ± 2.4 | 12.5 ± 2.2^a^ | 10.1 ± 2.4^b^ | 12.2 ± 2.0^a^ | 0.001 |
| Hematocrit, *mmol/L* | 35.4 ± 7.3 | 36.9 ± 6.7^a^ | 30.0 ± 7.5^b^ | 36.0 ± 5.8^a^ | 0.001 |
| Mean corpuscular volume, *fL* | 83.5 ± 6.5 | 82.9 ± 4.9 | 85.4 ± 10.5 | 83.9 ± 4.7 | 0.143 |
| Leukocytes, *10^3^/uL* | 8.4 ± 12.4 | 8.3 ± 3.8 | 7.9 ± 26.0 | 10.6 ± 8.4 | 0.740 |
| Erythrocytes, *10^6^/uL* | 4.2 ± 0.87 | 4.4 ± 0.77 | 3.4 ± 0.78 | 4.2 ± 0.70 | 0.001 |
| *Data are reported as mean ± standard deviation or median (percentile 25-75). Statistical analyses were performed by one-way ANOVA with a post-hoc Bonferroni’s multiple comparison tests. All results were considered statistically significant at p < 0.05. Differences among groups are indicated by letters, where a > b > c.* | | | | | |
